# Kinesio Taping®️ Plus Exercise Versus Sham Taping Plus Exercise and Exercise Alone for Lateral Epicondylitis: A Systematic Review and Meta-Analysis of Randomized Controlled Trials

**DOI:** 10.7759/cureus.100228

**Published:** 2025-12-27

**Authors:** Muhammad Tayyab, Zawar Ahmad, Rizwan Akbar, Muhammad Tanveer, Rahman Syed, Ameer Afzal Khan, Muhammad Younas Khan, Manahil Noor, Anfal Khan

**Affiliations:** 1 Trauma and Orthopaedics, Hayatabad Medical Complex Peshawar, Peshawar, PAK; 2 Trauma and Orthopaedics, Kettering General Hospital, Kettering, GBR; 3 Trauma and Orthopaedics, University College Hospital, London, GBR; 4 Trauma and Orthopaedics, Royal Stoke Hospital, Stoke on Trent, GBR; 5 Internal Medicine, Swat Medical College, Swat, PAK; 6 Internal Medicine, Saidu Medical College, Swat, PAK; 7 Orthopaedics, Saidu Teaching Hospital, Swat, PAK; 8 Medicine and Surgery, Gomal Medical College, DI Khan, PAK

**Keywords:** kinesio taping, lateral epicondylitis, physical therapy, systematic review and meta analysis, tennis elbow

## Abstract

Lateral epicondylitis (LE), commonly known as tennis elbow, is a frequent overuse injury of the wrist extensor tendons that leads to pain, weakness, and impaired upper-limb function. Kinesio Taping®️ (KT) (Kinesio Holding Corporation, Albuquerque, USA) has been increasingly used as an adjunct to exercise therapy, but its clinical efficacy remains uncertain. This systematic review and meta-analysis aimed to evaluate the effectiveness of KT combined with exercise compared with sham taping plus exercise and exercise alone in patients with lateral epicondylitis. Following the Preferred Reporting Items for Systematic Reviews and Meta-Analyses (PRISMA) guidelines, a comprehensive search was conducted in PubMed, Google Scholar, Cochrane Library, ClinicalTrials.gov, and Semantic Scholar up to October 23, 2025. The review protocol was registered in the International Prospective Register of Systematic Reviews [PROSPERO; ID: (CRD420251179581)]. Randomized controlled trials (RCTs) assessing KT plus exercise against sham taping or exercise alone were included. Data were pooled using a random-effects model, and results were expressed as mean differences (MD) with 95% confidence intervals (CI). Seven RCTs involving 271 participants were analyzed. KT combined with exercise significantly reduced pain compared with sham taping plus exercise (MD = -1.71; 95% CI: -3.31 to -0.10) and exercise alone (MD = -1.39; 95% CI: -2.63 to -0.14). Functional improvement assessed by the patient-rated tennis elbow evaluation (PRTEE) was also greater in the KT group for both comparisons (MD = -15.6 and -29.15, respectively). Grip strength improved by 3.96 kg (95% CI: 0.41-7.51) with KT plus exercise. In conclusion, KT used alongside exercise provides superior short-term improvements in pain, function, and grip strength compared with sham taping or exercise alone. Further standardized, long-term trials are warranted to confirm these findings.

## Introduction and background

Lateral epicondylitis (LE), also known as "tennis elbow," is a common overuse injury to the forearm extensor tendons that causes pain and tenderness across the humerus' lateral epicondyle [1]. It affects about 1-3% of adults, especially those who participate in repetitive wrist extension or gripping activities, resulting in significant pain, disability, and productivity loss [2,3]. The underlying pathology is microtears and degenerative alterations of the extensor carpi radialis brevis tendon, rather than inflammation [4]. Conservative management, which includes rest, non-steroidal anti-inflammatory drugs (NSAIDs), bracing, and physical therapy, remains the primary therapeutic strategy [5]. Eccentric strengthening and stretching activities are effective rehabilitation treatments for regaining function and reducing pain [6]. However, a fraction of patients experience chronic symptoms despite standard therapy, motivating the investigation of supplementary modalities, such as Kinesio Taping®️ (KT) (Kinesio Holding Corporation, Albuquerque, USA).

KT, introduced by Kenzo Kase in the 1970s, is an elastic therapeutic tape that supports muscles and joints without restricting mobility [7]. Proposed mechanisms include increased proprioception, improved circulation, and enhanced pain management through cutaneous stimulation [8]. While KT has gained popularity for a variety of musculoskeletal problems, the evidence for its effectiveness in LE is inconsistent. Some randomized controlled studies (RCTs) indicate that KT paired with exercise may improve pain alleviation and grip strength [9,10]. In contrast, others reported no significant effect compared to sham taping or exercise alone [11].

Previous reviews of KT across many musculoskeletal diseases lacked condition-specific subgroup analysis or focused comparisons to exercise-based rehabilitation [6]. Therefore, this systematic review and meta-analysis aimed to synthesize current evidence from RCTs comparing the effects of KT plus exercise versus sham taping plus exercise and exercise alone in improving pain, function, and grip strength among patients with lateral epicondylitis.

## Review

Methodology

This meta-analysis was conducted following the Preferred Reporting Items for Systematic Reviews and Meta-Analyses (PRISMA) guidelines [12]. The study adhered to the principles of the Declaration of Helsinki and was exempt from Institutional Review Board (IRB) approval as it involved secondary analysis of published studies. The review protocol was registered in the International Prospective Register of Systematic Reviews [PROSPERO; ID: (CRD420251179581)].

Search Strategy

A comprehensive literature search was performed in PubMed, Google Scholar, Semantic Scholar, ClinicalTrials.gov, and the Cochrane Library from database inception to 23 October 2025, without language restrictions. The search combined both MeSH terms and free-text keywords related to kinesio taping and lateral epicondylitis. The primary search string was:

("kinesio taping" OR "kinesiotape" OR "kinesio tape" OR "elastic therapeutic tape") AND ("lateral epicondylitis" OR "tennis elbow") AND (exercise OR physiotherapy OR rehabilitation) AND (randomized OR RCT OR "controlled trial").

Additional searches used combinations such as “sham taping,” “placebo taping,” and “kinesio taping plus exercise” to identify relevant randomized trials. Reference lists of included articles and prior reviews were manually screened for additional eligible studies. Duplicates were removed before screening.

Study Selection

Two investigators independently screened titles and abstracts for relevance, followed by full-text assessment against inclusion criteria. Disagreements were resolved by discussion with a third reviewer. Studies were included if they met all of the following criteria: 1) Randomized controlled trials (RCTs) (non-crossover). 2) Participants diagnosed clinically with lateral epicondylitis (tennis elbow). 3) Intervention: KT in combination with exercise, physiotherapy, or stretching. 4) Comparator: sham (placebo) taping plus exercise or exercise alone. 5) Reported at least one quantitative outcome on pain intensity [vascular analog scale (VAS) [13], numeric rating scale (NRS) [14], or function patient-rated tennis elbow evaluation (PRTEE) [15], quick-disabilities of the arm, shoulder, and hand questionnaire (Q-DASH) [16], short form-36 health survey(SF-36) [17], grip strength [18]). 6) Reported mean ± SD for both intervention and control groups at baseline and post-intervention. 7) Published in peer-reviewed journals and available in full text.

Exclusion criteria were: (1) Non-randomized, observational, or quasi-experimental designs; (2) Animal or pediatric studies; (3) Conference abstracts without full data; (4) Trials lacking clear comparator arms or missing numerical outcome data; (5) Studies assessing other modalities [e.g., dry needling, laser, or extracorporeal shock wave therapy (ESWT)] without a KT arm.

Data Extraction

Data were extracted independently by two reviewers using a standardized form. Extracted information included: author and year, study design, sample size, participant demographics, intervention and comparator descriptions (including tape technique, duration, and exercise protocol), follow-up period, and outcomes measured through VAS [13], NRS, PRTEE [15], Q-DASH [16], grip strength [18], SF-36 [17], Cyriax resisted muscle test [19]. Both pre- and post-intervention mean values and standard deviations were extracted whenever available, and change scores were calculated accordingly. Discrepancies were resolved by consensus or by consulting a third reviewer. When required data were missing, study authors were contacted via email.

Risk of Bias and Study Quality Assessment

Risk of bias for each RCT was evaluated independently by two reviewers using the Cochrane RoB 2 tool [20]. The following domains were assessed: (1) random sequence generation, (2) allocation concealment, (3) blinding of participants and assessors, (4) incomplete outcome data, (5) selective reporting, and (6) other potential biases. Each study was rated as low, some concerns, or high risk of bias.

Outcomes

The primary outcomes were: 1) Pain reduction measured by the VAS [13] or NRS [14]. 2) Functional improvement measured by the PRTEE [15]. Secondary outcomes included: 1) Grip strength improvement. 2) Disability score changes using Quick-DASH [16] or SF-36 [17]. 3) Cyriax resisted muscle test (CRMT) [19] results when reported.

Statistical Analysis

Data synthesis and statistical computations were performed using Review Manager (RevMan, version 5.4, The Cochrane Collaboration, Copenhagen, Denmark) and Meta-Essentials (Erasmus Research Institute of Management, Rotterdam, The Netherlands). Continuous outcomes were pooled as mean differences (MD) with 95% confidence intervals (CIs). Heterogeneity was assessed using the I² statistic, with <25% considered low, 25-75% moderate, and >75% high heterogeneity. Tau² and Chi² tests were used to evaluate model fit. A random-effects model (DerSimonian-Laird) was employed due to expected inter-study variability in taping techniques and exercise protocols. Meta-analyses were not performed for the CRMT and SF-36 outcomes because of limited data availability and heterogeneity in outcome reporting across studies.

For trials with three arms (KT + exercise, sham + exercise, exercise alone), the shared control groups were divided evenly to prevent double-counting in pairwise comparisons. Primary comparisons were: (1) KT + exercise vs sham + exercise (specific effect); (2) KT + exercise vs exercise alone (pragmatic effect).

Sensitivity analyses were performed using a ‘leave-one-out’ approach to test the stability of pooled results. The results of these analyses showed that no single study substantially altered the overall effect estimates, confirming the robustness of the findings. Subgroup analyses were planned based on comparator type (sham vs exercise alone), follow-up duration, and pain assessment scale.

Results

**Figure 1:**
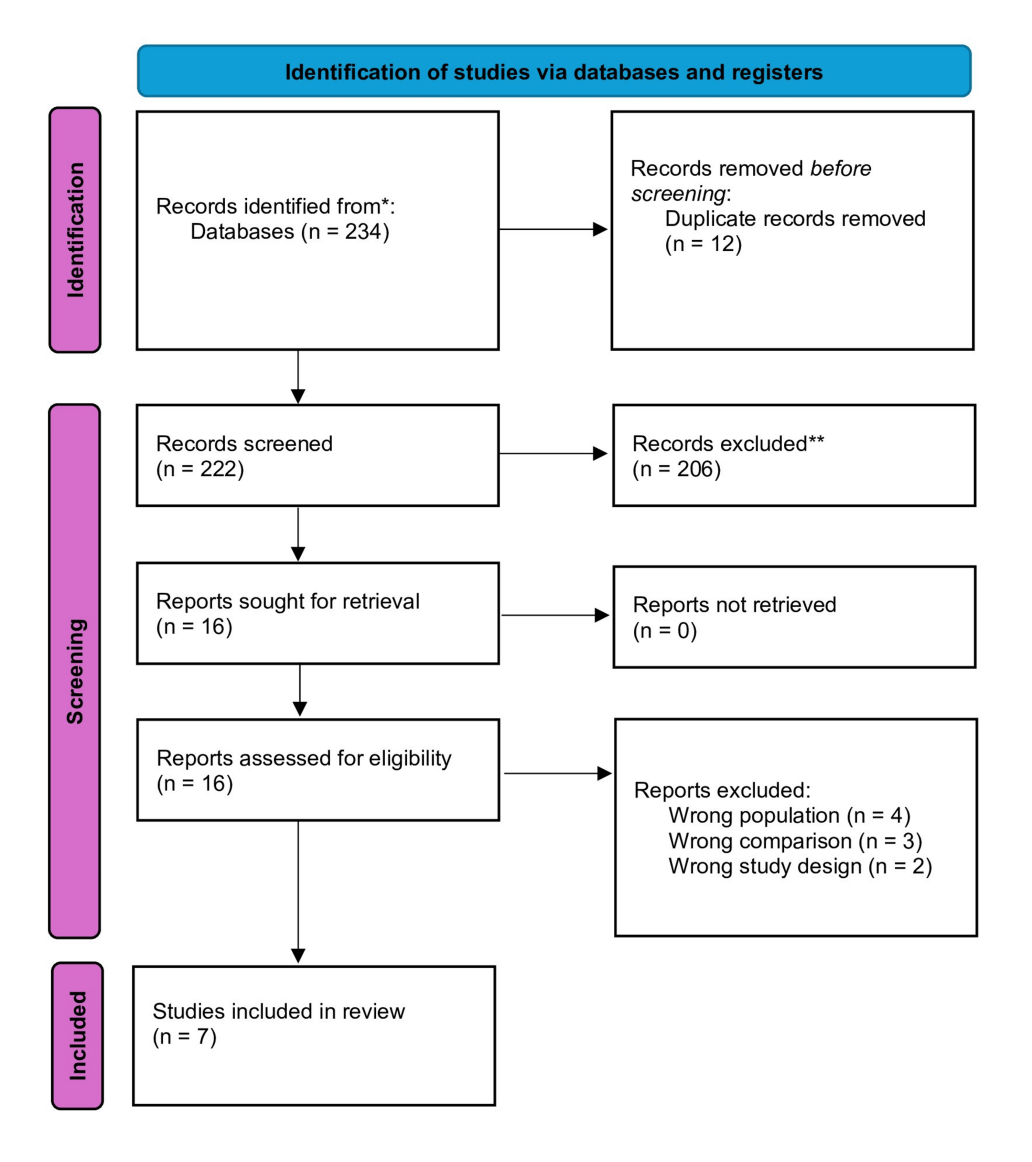
PRISMA flow diagram of study selection for systematic review and meta-analysis. PRISMA [12].

A total of seven RCTs [9-11, 21-24] were included in this meta-analysis, with (n=271) participants in the studies. Figure 1 shows the PRISMA flow diagram for study selection.

The studies used KT plus exercise, Sham taping plus exercise, and exercise alone as interventions with three to four weeks of follow-up. The study characteristics are summarized in Table 1.

**Table 1:** Characteristics of included studies. RCT: randomized controlled trial; KT: Kinesio Taping®️; E: exercise; ST: sham taping; VAS: visual analogue scale [13]; NRS: numeric rating scale [14]; PRTEE: patient-rated tennis elbow evaluation [15]; Q-DASH: quick-disabilities of the arm, shoulder, and hand questionnaire [16]; SF-36: short form-36 [17]; Grip strength [18]; CRMT: Cyriax resisted muscle test [19].

Author	Design	Intervention	Control	Sample size	Outcomes	Follow-up
Balevi et al. [9]	RCT	KT + Stretching+ Exercise	Sham taping + Stretching	KT+E = 25, ST+E=25	NRS [14], PRTEE [15], Cyriax resistant muscle test, SF-36	6 week
Yüksel et al. [21]	RCT	KT + therapeutic Exercises	Therapeutic exercises	KT+E=18, Exercise=17	VAS [13], Q-DASH [16], PRTEE [15], Grip Strength [18]	4 week
Altas et al. [22]	RCT	KT and performed Exercise	Exercise	KT + E=26, E=26	VAS [13], DHI, PRTEE [15], DASH [16], Grip Strength [18]	3, 24 week
Akkurt et al. [10]	RCT	KT + Exercise	Sham taping+ exercise	KT+E=21, ST+E=21	VAS [13], PRTEE [15], DASH [16], SF36 [17]	3, 7 week
Abdelmonem et al. [23]	RCT	KT + Physiotherapy	Physiotherapy	KT+E=15, E=15	VAS [13]	4 weeks
Eraslan et al. [24]	RCT	KT + Physiotherapy	Physiotherapy	KT+E=15, E=17	VAS [13], PRTEE [15], CRMT [19], Grip, Grip Strength [18]	3 weeks
Giray et al. [11]	RCT	KT + Exercise	Sham taping + Exercise	KT+E=10, ST+E=10, E=10	VAS [13], PRTEE [15], Grip, Q-DASH [16], Grip Strength [18]	4 weeks

Pain at Rest (VAS)

**Figure 2:**
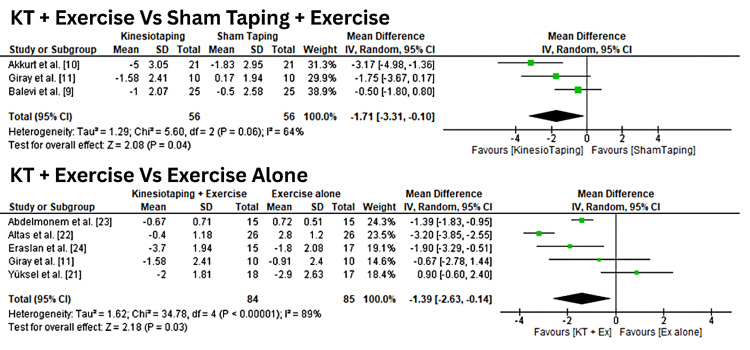
Forest plots comparing the effects of KT plus exercise versus control interventions (sham taping and exercise alone) on pain at rest (VAS). VAS: visual analog scale [13]. KT: Kinesio Taping®️.

Across studies, KT combined with exercise significantly reduced resting pain compared with sham taping plus exercise, with a mean difference of -1.71 (95% CI: -3.31 to -0.10). When compared with exercise alone, KT plus exercise also showed superior pain reduction, with a mean difference of -1.39 (95% CI: -2.63 to -0.14) at four weeks, as shown in Figure 2. The observed heterogeneity may be attributable to differences in participant characteristics, KT application techniques, exercise protocols, and follow-up durations. A “leave-one-out” sensitivity analysis was performed to test the stability of these results, confirming that no single study disproportionately influenced the pooled estimates.

Functional Status (PRTEE)

**Figure 3:**
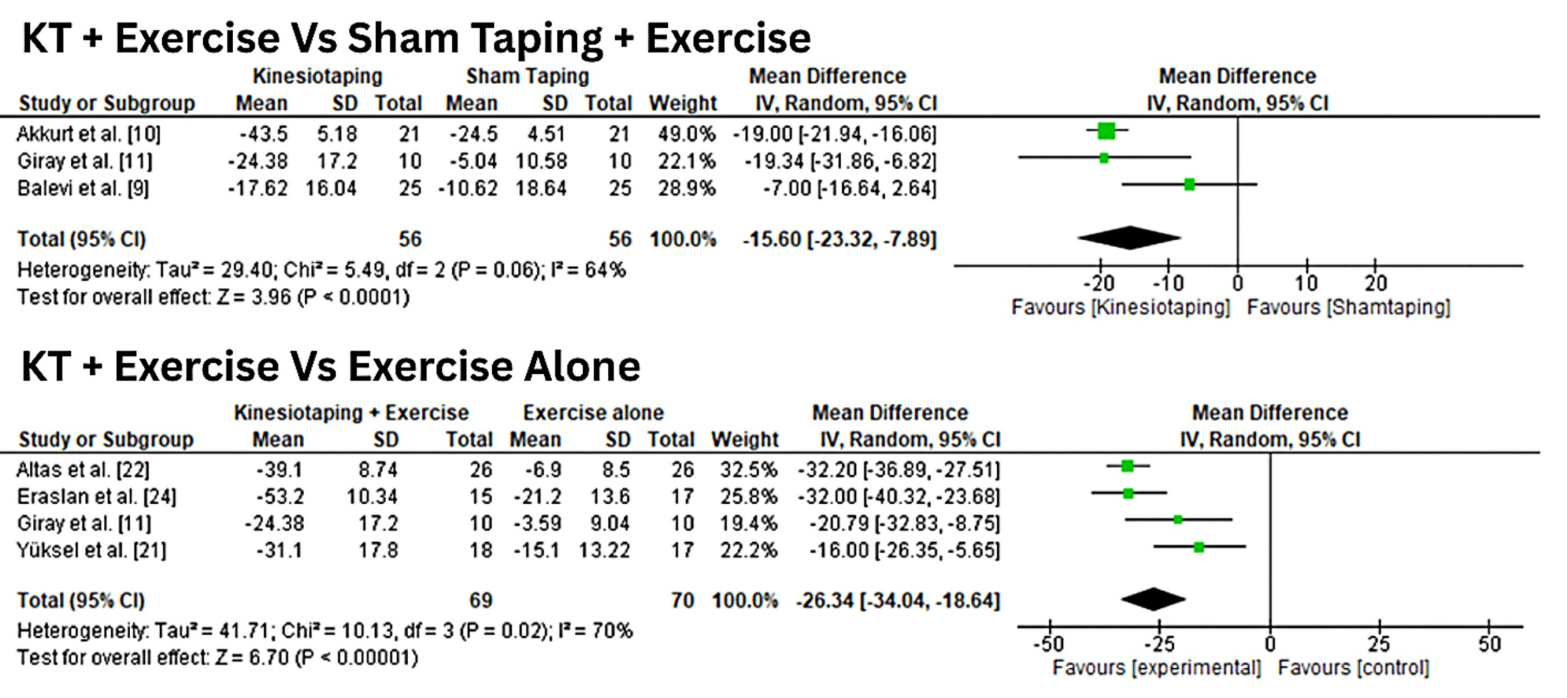
Forest plots comparing the effects of KT plus exercise versus control interventions (sham taping and exercise alone) on functional outcomes measured by the PRTEE. PRTEE: patient-rated tennis elbow evaluation [15]. KT: Kinesio Taping®️.

Consistent with the pain outcomes, KT significantly improved Patient-Rated Tennis Elbow Evaluation (PRTEE) [15] scores. The pooled mean reduction was -15.6 (95% CI: -23.32 to -7.89; I² = 64%) for KT plus exercise versus sham taping plus exercise. Compared with exercise alone, the mean reduction in PRTEE was even greater, -26.34 (95% CI: -34.04 to -18.64; I² = 70%), as shown in Figure 3. The heterogeneity observed across studies may be related to differences in KT application techniques, exercise protocols, participant characteristics, and follow-up durations. Sensitivity analyses using a ‘leave-one-out’ approach were performed, showing that no single study unduly influenced the pooled results. 

Grip Strength

**Figure 4:**
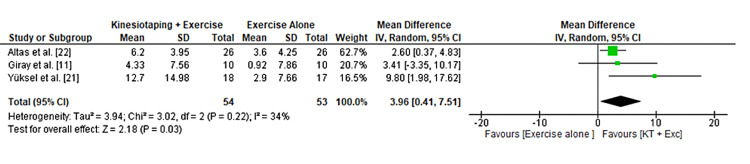
Forest plot displaying the effect of KT on grip strength improvement compared with control interventions. grip strength [18]. KT: Kinesio Taping®️.

KT application produced a significant improvement in grip strength, with a pooled mean difference of 3.96 (95% CI: 0.41 to 7.51) and moderate heterogeneity (Figure 4). While some studies reported no intergroup differences in elbow flexion and extension strength, the overall trend favored KT for functional recovery.

Upper Limb Function (Q-DASH)

**Figure 5:**
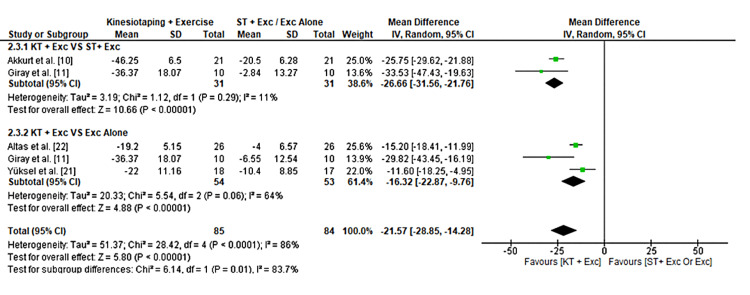
Forest plot comparing the impact of KT on upper limb function measured by Quick Disabilities of the Arm, Shoulder, and Hand (Q-DASH) scores. Quick-DASH [16]. KT: Kinesio Taping®️.

The pooled mean reduction in Q-DASH scores was -21.57 (95% CI: -28.85 to -14.28), indicating functional improvement. The effect was more pronounced when comparing KT versus sham taping (-26.66 [95% CI: -31.56 to -21.76]) than versus exercise alone (-16.32 [95% CI: -22.87 to -9.76]), as shown in Figure 5. This subgroup difference suggests that KT may provide additional benefit over sham taping, whereas the incremental benefit over exercise alone is smaller. Possible reasons for this difference include variations in participant characteristics, exercise protocols, and KT application techniques across studies, warranting cautious interpretation of subgroup effects.

Cyriax Resisted Muscle Test (CRMT)

Within-group analyses showed significant improvement in CRMT [20] outcomes, particularly in elbow pronation and grip strength of elbow extension (p = 0.002 and p = 0.001, respectively) [9]. Although improvements were noted in all groups, the KT group showed comparable or superior gains compared to other interventions [24].

Quality of Life (SF-36)

Both the KT and control groups demonstrated significant improvements in SF-36 [17] quality of life domains. However, the KT group showed greater gains in six of eight subscales-excluding physical and social functioning-indicating a broader enhancement in patient well-being (p < 0.05) [10]. The improvement in SF-36 persisted up to six weeks post-treatment [9]. 

Risk of Bias Assessment

**Figure 6:**
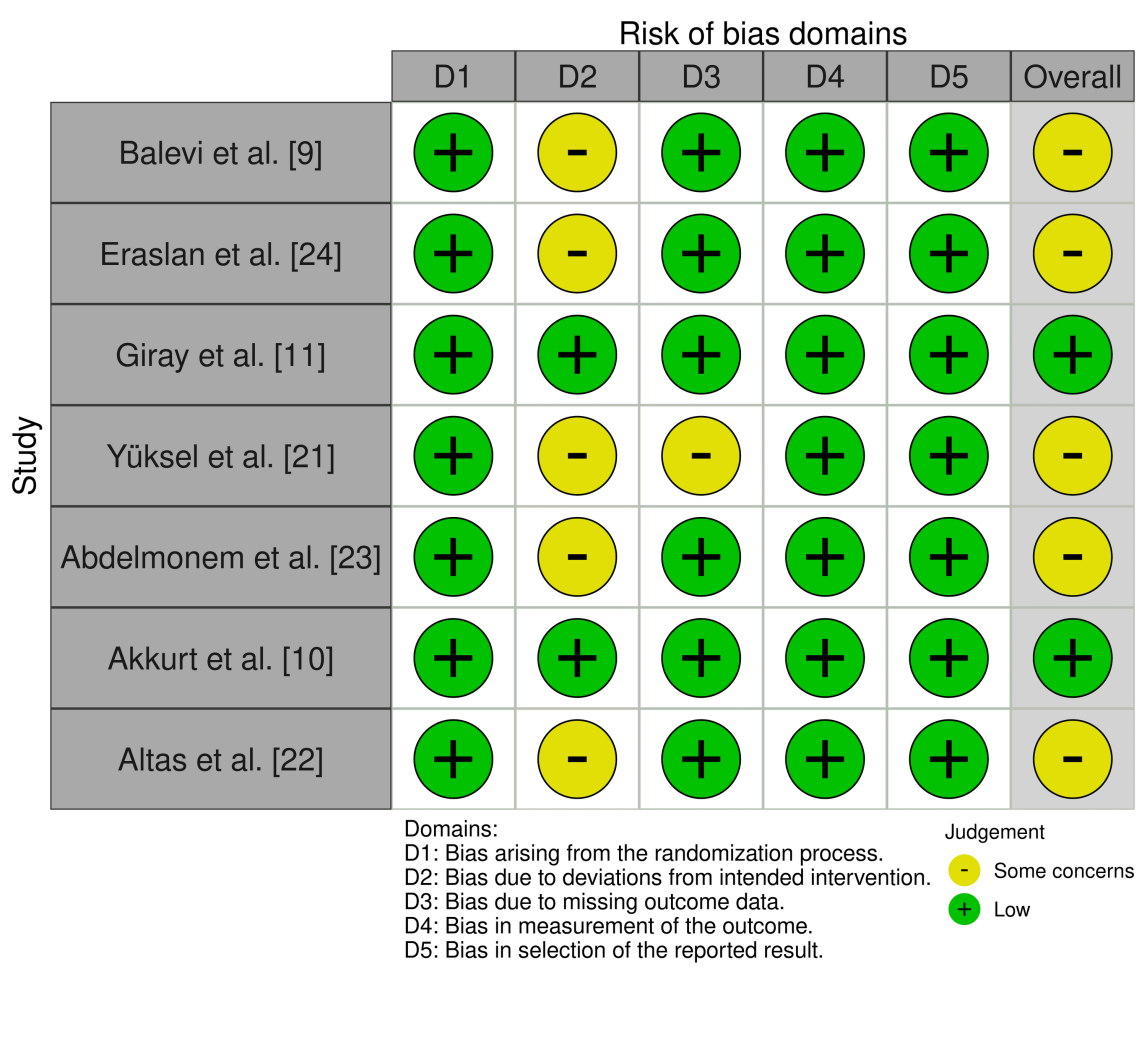
Risk of bias summary of included randomized controlled trials assessed using the Cochrane RoB 2 tool. Cochrane RoB 2 [20].

The overall methodological quality of the included trials was high. Two studies, Giray et al. and Akkurt et al. [10,11], were rated as low risk of bias, while the remaining five [9,21-24] had some concerns, primarily due to limited participant blinding or incomplete adherence data (Figure 6). No study was rated as high risk, indicating a generally robust evidence base.

Discussion

This systematic review and meta-analysis synthesized evidence from seven randomized controlled trials evaluating the effectiveness of KT combined with exercise compared to sham taping plus exercise and exercise alone in patients with LE. The polled results showed that using KT as an addition to exercise therapy resulted in significant improvements in pain reduction (VAS/NRS) [13,14], functional recovery (PRTEE) [15], and grip strength [18] when compared to both control conditions. These findings suggest that KT may provide additional short-term benefits in the therapy of LE compared to traditional rehabilitation exercises.

The current findings are consistent with earlier research showing that KT can improve pain alleviation and function in a variety of musculoskeletal conditions, including shoulder impingement and patellofemoral pain [25-27]. Balevi et al. [9] and Akkurt et al. [10] found significant improvements in pain and PRTEE scores with KT application in chronic LE, which is consistent with our pooled analysis that found greater decreases in pain intensity and functional impairment. Giray et al. [11] observed no significant difference between KT and exercise alone, which could be due to the shorter follow-up period and changes in taping method and exercise regimes. Methodological heterogeneity is likely responsible for the moderate-to-high I² values seen across analyses.

KT's analgesic action can be explained using a variety of hypothesised methods. The application of elastic tape may stimulate cutaneous mechanoreceptors, altering afferent information to the central nervous system and lowering pain through the gate control theory [7]. Furthermore, KT may increase local circulation, lymphatic drainage, and proprioceptive feedback, resulting in faster muscle recovery and a reduction in inflammation-related metabolites [8,28]. These mechanisms, when paired with structured exercise programs, may synergistically improve neuromuscular control and tendon healing, explaining the reported functional benefits.

The improved grip strength found in the KT group adds to its biomechanical benefits. By providing external support to the wrist extensors and improving joint alignment, KT may alleviate excessive strain on the extensor carpi radialis brevis tendon during resisted extension [29]. This functional advantage was also reflected in higher Q-DASH and SF-36 scores in the intervention groups, indicating a more general improvement in upper limb performance and quality of life. Although KT improved grip strength, the effect size appears small to moderate relative to clinical thresholds for meaningful change in lateral epicondylitis. This suggests that KT provides supportive not major biomechanical benefits. 

Despite these encouraging results, several limitations warrant consideration. First, the number of included trials and their sample sizes were modest, potentially limiting statistical power. Second, variability in taping methods (Y-strip vs. I-strip, tension levels), exercise protocols, and follow-up durations introduces heterogeneity that could influence effect estimates. Third, most studies evaluated short-term outcomes (three to seven weeks), with limited data on the durability of KT’s effects. Fourth, blinding participants to KT is inherently challenging due to its visible and tactile nature, introducing potential performance bias. Finally, publication bias cannot be entirely excluded given the small number of studies per comparison group.

Nonetheless, this meta-analysis presents the most thorough quantitative evidence to date on the use of KT and exercise for LE. The findings highlight KT's potential as a safe, cost-effective, and simply implementable supplementary therapy for improving pain alleviation and function throughout rehabilitation. Future research should prioritize standardizing KT application techniques, incorporating instrumented biomechanical assessments, and evaluating the persistence of benefits after treatment ends. Direct comparisons of different KT configurations (e.g., facilitatory vs. inhibitory taping) are also warranted. Additionally, pragmatic clinical trials would help determine the real-world effectiveness and applicability of KT in routine physiotherapy settings.

## Conclusions

This meta-analysis indicates that KT used alongside exercise therapy significantly improves short-term pain relief, grip strength, and functional outcomes in patients with lateral epicondylitis compared to exercise or sham taping alone. KT appears to enhance rehabilitation through improved proprioception, muscle activation, and tendon unloading. However, the benefits seem primarily short-term, and evidence regarding long-term effects remains limited. Differences in taping techniques, intervention protocols, and assessment tools contribute to heterogeneity across studies. Overall, KT is a safe, cost-effective, and supportive adjunct to conventional physiotherapy, but further high-quality randomized trials with standardized methodologies and extended follow-up are required to confirm its sustained efficacy and establish optimal application protocols.
